# Achieving Optimal Short- and Long-term Responses to Paediatric Growth Hormone Therapy

**DOI:** 10.4274/jcrpe.galenos.2019.2019.0088

**Published:** 2019-11-22

**Authors:** Jan M. Wit, Asma Deeb, Bassam Bin-Abbas, Angham Al Mutair, Ekaterina Koledova, Martin O. Savage

**Affiliations:** 1 Leiden University Medical Centre, Department of Paediatrics, Leiden, Netherlands; 2 Mafraq Hospital, Clinic of Paediatric Endocrinology, Abu Dhabi, United Arab Emirates; 3 King Faisal Specialist Hospital and Research Center, Department of Paediatrics, Riyadh, Saudi Arabia; 4 King Abdullah Specialised Children’s Hospital, Ministry of National Guard Health Affairs, Riyadh, Saudi Arabia; 5 King Abdullah International Medical Research, Clinic of Paediatrics, Riyadh, Saudi Arabia; 6 King Saud bin Abdulaziz University for Health Science, Clinic of Paediatrics, Riyadh, Saudi Arabia; 7 Global Medical Affairs Endocrinology, Merck KGaA, Darmstadt, Germany; 8 William Harvey Research Institute, Barts and the London Faculty of Medicine and Dentistry, London, United Kingdom

**Keywords:** Paediatrics, short stature, growth hormone therapy, growth hormone deficiency, Turner syndrome, small for gestational age

## Abstract

It is over sixty years since the first administration of human growth hormone (GH) to children with GH deficiency, and over thirty years since recombinant human GH has been available for treatment of GH deficiency and a wider range of non-GH deficiency disorders. From a diagnostic perspective, genetic analysis, using single gene or Sanger sequencing and more recently next generation or whole exome sequencing, has brought advances in the diagnosis of specific causes of short stature, which has enabled therapy to be targeted more accurately. Genetic discoveries have ranged from defects of pituitary development and GH action to abnormalities in intracellular mechanisms, paracrine regulation and cartilage matrix formation. The strategy of GH therapy using standard doses has evolved to individualised GH dosing, depending on diagnosis and predictors of growth response. Evidence of efficacy of GH in GH deficiency, Turner syndrome and short children born small for gestational age is reviewed. The importance of critical assessment of growth response is discussed, together with the recognition and management of a poor or unsatisfactory growth response and the organisational issues related to prevention, detection and intervention regarding suboptimal adherence to GH therapy.

## Introduction

**Figure 1:**
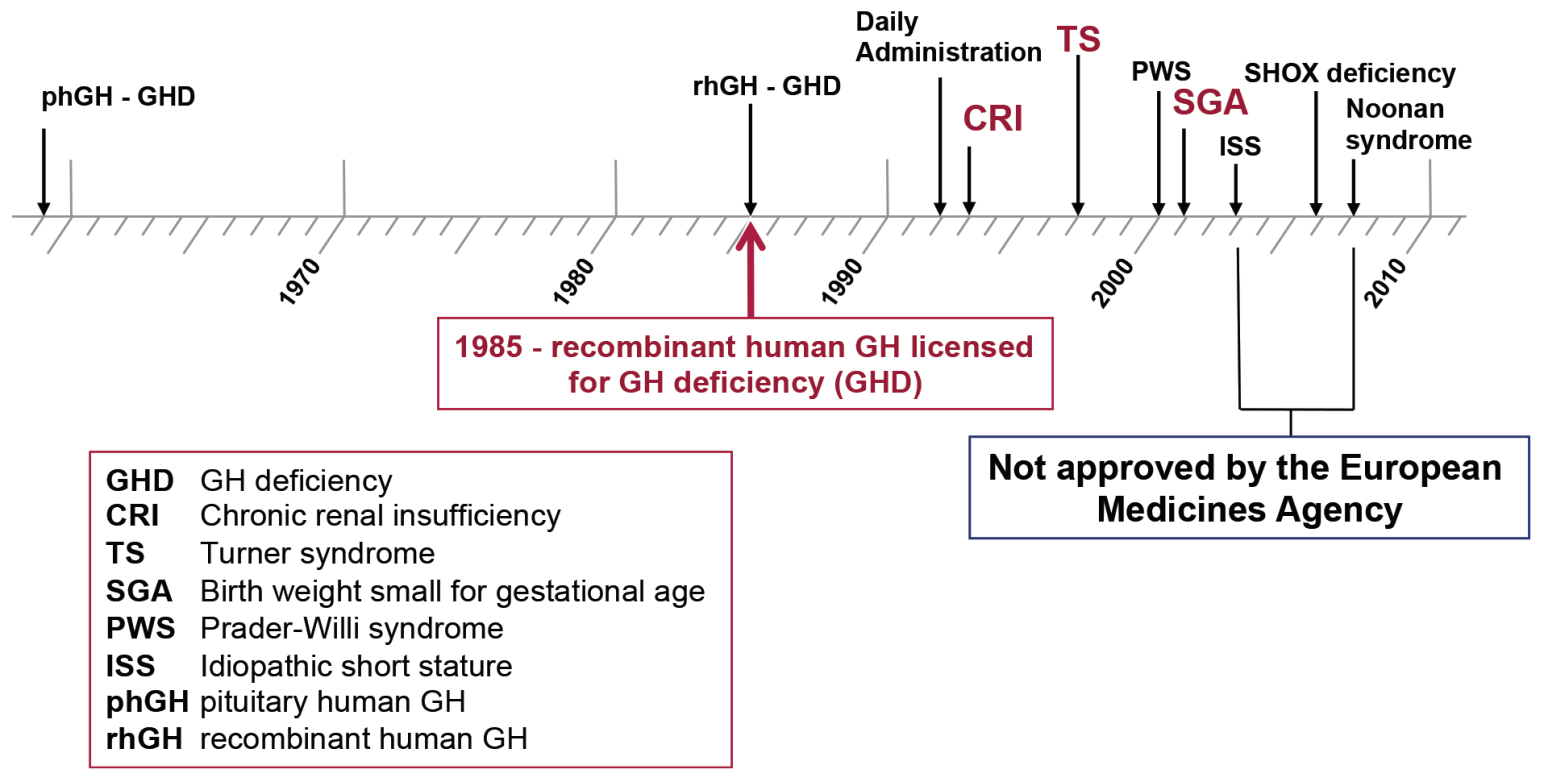
Growth disorders with approval for growth hormone therapy from the United States Food and Drug Administration and the European Medicines Agency

Human pituitary-derived growth hormone (GH) has been in use to promote growth in short children for more than 25 years, until it was halted in 1985 due to recognition of the association with Creutzfeldt-Jakob disease (1). The first recombinant human GH received approval for paediatric clinical use for growth promotion in 1985, from both the United States Food and Drug Administration (FDA) and the European Medicines Agency (EMA). Initially, the approval was specifically for children with GH deficiency (Figure 1), but over time GH has been licensed for use in a number of non-GH deficiency growth disorders, including chronic renal insufficiency, Turner syndrome and short stature related to birth size in small for gestational age (SGA) children. The non-GH deficiency disorders of Noonan syndrome and idiopathic short stature have also received approval for GH use from the FDA, but not the EMA, and short stature due to *SHOX* gene haploinsufficiency and Prader-Willi syndrome (PWS) have received approval for some GH formulations in some countries (2,3,4).

The capacity to secrete endogenous GH and the sensitivity to administered GH vary greatly, both within and among these disorders (5). Thus, there is a continuum whereby GH secretion is very low and responsiveness to treatment is high in patients with severe GH deficiency, in contrast to those with severe GH resistance (Laron syndrome) where GH secretion is high and response to administered GH is very low or non-existent (5,6,7). Conditions in-between include PWS and GH neurosecretory dysfunction, with good GH sensitivity but diminished secretion and idiopathic short stature, chronic renal insufficiency and SGA, with slightly reduced or normal GH secretion and variable GH responsiveness. When GH sensitivity is decreased, particularly in patients with SGA, Turner syndrome and idiopathic short stature, higher or pharmacological GH doses are generally required (5,8).

The differences in sensitivity to administered GH in children with growth failure arising from different conditions means that treatment management varies according to factors including the diagnosis, gender and age of the patient at GH initiation. An Advisory Board was convened in Dubai in December 2017, by Merck Serono Middle East FZ-LLC, Dubai, United Arab Emirates, with the aim of addressing the issues of the short- and long-term management of paediatric GH therapy for children with growth failure due to different conditions. This article reports the discussions and conclusions of the Advisory Board meeting.

## The Genetic Basis of Short Stature

Human adult height is a polygenic trait in which the additive genetic contribution to normal variation is reported to be approximately 80% (9,10). While it is polygenic overall, multiple monogenic defects in genes coding for proteins with key functions in GH secretion and action have been identified to be associated with growth failure. At least eight genes have been identified in which individual abnormalities were associated with isolated GH deficiency and at least 19 genes where mutations resulted in combined pituitary hormone deficiency, with several related to specific syndromes (11,12). There were also 10 genes reported to be associated with GH resistance or insulin-like growth factor-1 (IGF-1) insensitivity. However, genome-wide association studies have indicated that only a minority of genes related to adult height are directly associated with the GH-IGF-1 axis and many genes act through other pathways (10).

Bone growth in the epiphyseal growth plate is influenced by many factors, including cytokines, nutritional status, other hormones such as thyroxine, glucocorticoids and gonadotropins, various paracrine factors within the extracellular matrix, and intracellular proteins (13,14). Next-generation sequencing has also shown that many genetic disorders that were previously thought to be only associated with skeletal dysplasia can present as dominant forms of apparent idiopathic short stature. These include, for example, abnormalities in the gene for the retinoic acid degrading enzyme *CYP26C1* (15), coding and non-coding regions of the short-stature homeobox-containing gene *SHOX* (16,17), the *ACAN* gene coding for the growth plate extracellular matrix proteoglycan aggrecan (18,19), the natriuretic peptide receptor-B gene *NPR2* (20,21,22) and the gene encoding Indian Hedgehog (*IHH*) (23).

These studies have led to a new paradigm (Opinion box) in which regulation of the epiphyseal plate is recognised to be pivotal to human linear growth, with disorders of the GH-IGF-1 axis making a less important contribution to height (13). The distinctions between idiopathic short or tall stature and skeletal dysplasias have become blurred and it is now understood that a genetic mutation can lead to a spectrum of phen. Gain of function mutations of various genes can lead to tall stature, whereas mild polymorphisms that modulate function and/or expression may result in low normal height. Mutations of genes that are not critical for growth, and mutations that are heterozygous or merely impair function of the gene product, can lead to isolated short stature, while mutations that cause severe loss of function affect critical genes causing skeletal dysplasias concomitant with growth failure (13,24). Therefore, testing for genetic defects in order to provide a diagnosis needs to be very specific and directed (25,26,27,28), although there are also arguments in favour of a hypothesis-free approach, using growth-specific whole exome sequencing-based gene panels (29,30,31).

## GH Therapy for GH Deficiency and Non-GH Deficiency Causes of Short Stature

### GH Therapy: Aims and Growth Response

For paediatric patients who receive exogenous GH for short stature, the therapy must be effective and safe. The induced catch-up growth should increase the height standard deviation score (SDS), with the aim of achieving an adult height SDS close to the genetic target height SDS, based on mid-parental height (2,32). Reports have suggested an association of excessive IGF-1 levels with adult morbidity, with the results that the aim of therapy for patients with initially low serum IGF-1 SDS is to treat with a GH dose that results in an IGF-1 increase to within the normal range (33,34). However, in patients with non-GH-deficient causes of growth failure, and initially normal IGF-1 SDS, elevated IGF-1 levels associated with higher GH doses may be required to achieve an acceptable height gain (34,35,36). GH treatment should also be as patient-friendly as possible, with appropriate injection devices and low injection volumes (37). Additionally, GH treatment should be cost-effective (34,38,39), providing sufficient efficacy with the lowest dose, which requires a personalised approach to therapy (40,41).

A number of different measurements have been used to assess growth response to GH treatment (42). These include the change in height SDS and height velocity at yearly intervals after starting GH therapy. Catch-up growth may also be determined from change in height over the first two years of GH treatment or using mathematical models of height SDS (43). However, there is no consensus on the definition of a good indicator of response and, if a relatively low cut-off level is taken, more than 50% of patients may be defined as poor responders (6,42,44). There may be multiple factors that affect response, such as concomitant disease, unanticipated GH insensitivity or poor adherence with the treatment (43). Nevertheless, this depends to a large extent on the correct diagnosis, because the aetiology has a major impact on the response to paediatric GH therapy (45).

### Prediction of Response

A number of models have been devised to predict the growth response to GH therapy for various aetiologies (41,46,47,48,49,50,51). Using such models, the mean predicted height velocity with a standard dose of 0.3 mg/kg/week is approximately 2 cm/year greater for children with GH deficiency, compared with children born SGA and girls with Turner syndrome. The factors that influence the prediction of the first-year growth response for patients with GH deficiency, Turner syndrome and SGA are shown in Table 1. The primary influence for children with GH deficiency is severity of the condition, whereas the primary influence for girls with Turner syndrome and children born SGA is the dose of GH per kg body weight per week (6,52,53).

Predictive models can also be used for long-term growth response (54,55,56). Factors that influence adult height are shown in Table 2, including the calculated variation explained by all predictive factors combined for each diagnostic cause (54,57,58,59). Height at GH initiation and mid-parental target height has an impact on adult height for patients in each of the diagnostic categories. The first-year response to GH treatment was strongly correlated with gain to adult height and, therefore, change in height SDS should be formally assessed at the end of year one of GH therapy (58,59,60). These predictive factors can be used to tailor GH treatment even if a formal mathematical model is not used (Opinion Box). Alternatively, GH dose can be individualised by adjusting the dose according to serum IGF-1 level (41).

## GH Therapy in Specific Diagnoses

### GH Deficiency

GH deficiency may either be isolated or occur together with deficiency of other pituitary hormones; patients with multiple pituitary hormone deficiencies will need replacement of additional hormones at appropriate levels over time, which could have a further impact on their growth rate. The severity of the GH deficiency, determined from the peak GH concentration seen on provocation testing, has a strong influence on the response rate. Children with more severe disease and very low stimulated GH peak have a greater response to GH therapy (44,46,61). Initiation of GH at as young an age as possible (Opinion Box) is also a key factor for a good response in GH-deficient patients and GH treatment should start when the patient is still pre-pubertal (45,57).

### Turner Syndrome

Age at GH initiation is also strongly negatively correlated with growth response in girls with Turner syndrome (62,63). The genetic variability of girls with Turner syndrome results in marked differences among patients of different phenotypic characteristics (64). Dysmorphic features, such as webbed neck, cubitus valgus, shortening of the 4^th^/5^th^ metacarpal and lymphoedema, may often be identified early, particularly if the chromosomal abnormalities include defects of the *SHOX* gene (17). While growth failure with decreased adult height is reported to occur in at least 95% of Turner syndrome cases, diagnosis and GH initiation is often delayed (64,65). Appropriate screening criteria, particularly using country-specific reference standards, can enable better and more efficient identification of short stature and earlier initiation of GH therapy (65). Dose of GH is also an important influence on growth in Turner syndrome and the recommended dose is generally around 50 µg/kg/day (64), which is higher than for patients with GH deficiency (3,35).

### Short Stature Related to SGA

For children born SGA, approximately 90% will show catch-up growth within the first 2-3 years of life (66,67). However, GH therapy may be required for those children who do not show catch-up growth and can also improve body composition and metabolic health (67,68,69). Efficacy of GH treatment is greater if started at an early age (66) and height SDS was shown to be increased to a greater extent when patients at start of treatment were younger than four years compared with those over four years (70). However, treatment before four years of age is not recommended and not approved in Europe (3). The dose approved by the EMA is 33 µg/kg/day, although higher doses are approved by the FDA (3,71). While higher doses have been reported to provide better short-term efficacy, they are associated with supraphysiological IGF-1 levels and are, therefore, not recommended (71). Additionally, the increase in height gain with a higher dose was mainly in the short-term catch-up period and there was little additional benefit of a higher dose over the longer term at adult height (72).

## Management of Poor Growth Response

### Principles of Management

**Figure 2:**
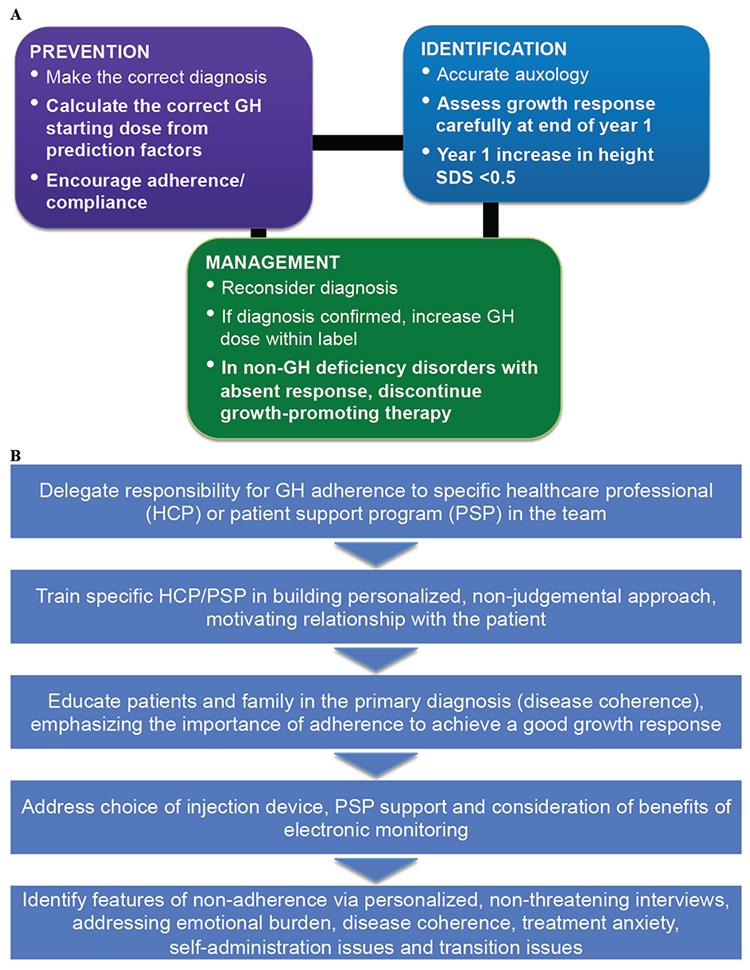
Management principles and strategy for patients with (A) a poor growth response and (B) non-adherence to GH therapy for short stature

The recognition of a poor growth response to GH treatment is an important part of management of children with growth failure. However, a poor growth response is reported surprisingly frequently, particularly in children with Turner syndrome or born SGA, but the reported incidence depends to a large extent on the criteria used (42). The principles for management of a poor response are summarised in Figure 2A. To prevent occurrence of a poor response, the diagnosis of the cause of growth failure must be correct and the dose of GH administered should be based on that diagnosis, preferably with a prediction of the anticipated response (41). Growth must be determined accurately and the response at the end of the first year should be assessed; if the response is insufficient the primary diagnosis should be ascertained and the dose checked to ensure appropriateness (6). If the response is very low, then discontinuing GH treatment should be considered, particularly for non-GH deficiency diagnoses. However, there is no consensus on the cut-off of a low or very low response (6). Our preference is to use the change in height SDS in pre-pubertal children and consider a first-year change <0.3 SDS to be insufficient. A poor response following confirmation of the diagnosis may require an increase in GH dose, although this should be within the approved label.

### Adherence to GH Therapy

The responsiveness of an individual patient to GH therapy can be determined from the difference between observed and predicted gain in height or height velocity (47). If responsiveness is reduced, while the diagnosis is deemed correct and presence of concomitant disease ruled out, then poor adherence to the treatment regimen should be considered (Opinion Box) (6,43,47,73). Monitoring of adherence should begin as soon as the treatment is initiated because some patients may not take the medication right from the start or renew their prescriptions, particularly if there is a lack of perceived need (74,75,76,77). While measurements of IGF-1 SDS may give an indication of adherence with GH, it is often not determined routinely and may not provide a definitive answer, because changes in concentration depend on multiple factors (77,78). Success of any therapy is dependent on good adherence and increased adherence may have a greater impact on health than improvements in specific medications. In the case of GH therapy, poor adherence has been shown to be associated with impaired clinical outcomes and reduced growth response (43,79,80,81).

Methods for assessing adherence have generally been poor, frequently relying on reporting by patients or carers, but have indicated that up to 82% of patients may miss at least some doses of GH (43,73,77). A strategy for prevention and management of adherence is outlined in Figure 2B. For effective management of poor adherence to GH, the paediatric endocrinologist or specialist nurse needs to learn techniques of non-judgemental motivational discussion. This requires time and organisation, knowledge of common issues affecting adherence at different treatment periods and ability to structure discussion with open questions, with emphasis on pre-GH treatment education. The same healthcare professional should discuss adherence at each outpatient visit. The strategy also involves addressing the choice of injection device and facility to identify adherence issues. New techniques of electronic monitoring are improving this process and provide important feedback data on evidence of sub-optimal adherence, which may not be available from self-reported data from patients and caregivers, clinical history or auxological measurements.

Electronic monitoring of GH injections is enabled through use of the easypod™ injection device and Easypod Connect^©^ system, which is the only such solution currently approved and with published information (43,73). The device facilitates administration of a pre-set dose of GH, automatically records injection times and doses, and provides the patient with information such as number of doses remaining (82). The injection information can be downloaded at any time via Easypod Connect by healthcare personnel, which enables distance monitoring, with less need for frequent face-to-face visits. Thus, healthcare personnel are able to address issues of non-adherence with treatment at an early stage before any decrease in GH efficacy. Studies to date have indicated good acceptance of the device and high levels of adherence over several years (83,84,85). Studies with the device have also shown a significant correlation of high adherence with improved outcomes (43,85).

### Manipulation of Puberty for Added Growth Advantage

Due to frequent delays in diagnosis of patients with short stature, GH treatment is often initiated close to, or even after, the start of the pubertal growth spurt. Studies have generally indicated that the growth response is greater if GH is started at a younger age, and particularly at the pre-pubertal stage, irrespective of the cause of short stature (45,57). While an increase in GH dose during puberty has been suggested, there are no clinical studies that have shown a convincing beneficial effect on adult height. Therefore, delaying puberty to allow exogenously administered GH to act for a longer period has been suggested as a strategy to improve overall linear growth (86). Oxandrolone administration has been examined in boys with constitutional delay of growth and idiopathic short stature, but had no significant effect on adult height (86,87). However, addition of oxandrolone to GH therapy has been studied in girls with Turner syndrome and provided approximately 3 cm of extra adult height gain (86,88).

Several small, off-label studies showed that delaying puberty with a gonadotropin-releasing hormone agonist (GnRHa) could increase adult height in children with idiopathic short stature or born SGA, but the effect was modest, not considered clinically significant and outweighed by adverse effects (89,90). When GH initiation is delayed and is close to or during puberty, adding a GnRHa may delay puberty and potentially prolong GH effectiveness. Although such combination treatment is not licensed, GnRHa added to GH has been examined in several studies in children with growth failure due to various different causes (86). The combination of a GnRHa with GH was reported to result in a significant increase in adult height in patients with GH deficiency (91,92) and also in those with idiopathic short stature (86,93) and born SGA (94). However, the GnRHa treatment has negative effects on body composition and, while these effects are reversible after GnRHa cessation, the effects on bone mineral density are of concern and could increase fracture risk (93,95,96).

## Conclusions

GH therapy for growth failure due to causes with approval for use to promote growth in short children can induce clinically beneficial short-term and long-term gains in height. The sensitivity and responsiveness to GH treatment are increased in children with GH deficiency compared with children with non-GH deficiency disorders, such as Turner syndrome or SGA. GH therapy should be individualised for each patient, based on the diagnosis and factors predicting growth response, such as age, severity of GH deficiency and deficit from genetic target height. A formal assessment of the response after the first year of GH therapy is recommended, with calculation of the gain in height SDS. The gain should ideally be compared with the individualised prediction; alternatively, cut-off levels of first-year height SDS change of 0.3 or 0.5 have been suggested as lower limits of an acceptable capacity for catch-up growth.

Good adherence to GH therapy is essential to achieve optimal short- and long-term responses, although management of poor adherence generally requires time and organisation. Novel techniques of electronic monitoring are helpful and can provide data that demonstrate when adherence is reduced, which may not be detectable from the patient’s history or auxological observations. In some clinical situations, such as GH deficiency, SGA with short stature at onset of puberty and idiopathic short stature, addition of a GnRHa (for a minimum of two years) to GH therapy can increase adult height gain, but adverse effects should be carefully monitored and a positive benefit-risk ratio has not been formally assessed by regulatory authorities; these combinations were used in clinical trials only and are not included in any GH therapy label at present.

## Figures and Tables

**Table 1:**
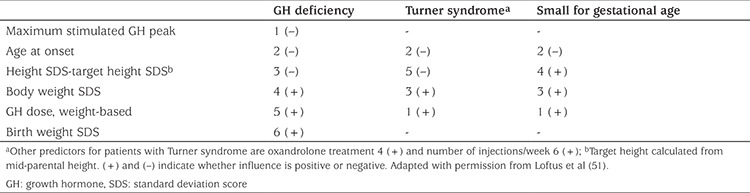
Predictive factors of the first year growth response to growth hormone treatment in patients with different causes of growth failure

**Table 2:**
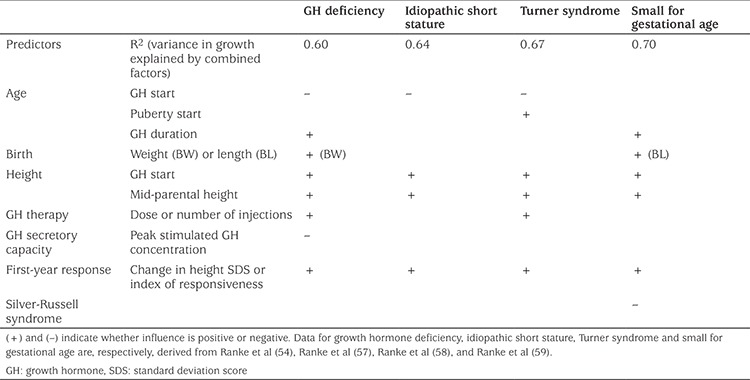
Factors predictive of near adult height with growth hormone treatment in patients with short stature due to various causes

**Opinion Box:**
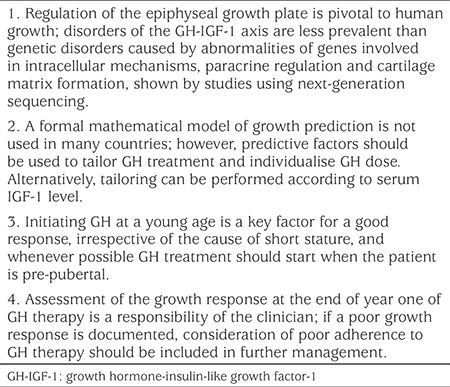
Consensus opinions of the advisory board
